# Factors influencing the cardiometabolic response to (poly)phenols and phytosterols: a review of the COST Action POSITIVe activities

**DOI:** 10.1007/s00394-019-02066-6

**Published:** 2019-09-06

**Authors:** Eileen R. Gibney, Dragan Milenkovic, Emilie Combet, Tatjana Ruskovska, Arno Greyling, Antonio González-Sarrías, Baujke de Roos, Francisco Tomás-Barberán, Christine Morand, Ana Rodriguez-Mateos

**Affiliations:** 1 UCD Institute of Food and Health, School of Agriculture and Food Science, University College Dublin, Dublin, Ireland; 2 INRA, UNH, Unité de Nutrition Humaine, CRNH Auvergne, Université Clermont Auvergne, Clermont-Ferrand, France; 3 School of Medicine, Dentistry and Nursing, College of Medical, Veterinary and Life Sciences, University of Glasgow, Glasgow, UK; 4 Faculty of Medical Sciences, University “Goce Delcev”-Stip, Štip, Republic of North Macedonia; 5 Unilever Research and Development Vlaardingen, Vlaardingen, The Netherlands; 6 Food and Health Lab. CEBAS-CSIC, Murcia, Spain; 7 The Rowett Institute, University of Aberdeen, Aberdeen, UK; 8 Department of Nutritional Sciences, School of Life Course Sciences, Faculty of Life Sciences and Medicine, King’s College London, London, UK

**Keywords:** Diet, Bioactive, Plant, Variation, Cardiometabolic diseases, Metabolism, Microbiome, Metabotype, Response, Gene expression, Health

## Abstract

**Purpose:**

Evidence exists regarding the beneficial effects of diets rich in plant-based foods regarding the prevention of cardiometabolic diseases. These plant-based foods are an exclusive and abundant source of a variety of biologically active phytochemicals, including polyphenols, carotenoids, glucosinolates and phytosterols, with known health-promoting effects through a wide range of biological activities, such as improvements in endothelial function, platelet function, blood pressure, blood lipid profile and insulin sensitivity. We know that an individual’s physical/genetic makeup may influence their response to a dietary intervention, and thereby may influence the benefit/risk associated with consumption of a particular dietary constituent. This inter-individual variation in responsiveness has also been described for dietary plant bioactives but has not been explored in depth. To address this issue, the European scientific experts involved in the COST Action POSITIVe systematically analyzed data from published studies to assess the inter-individual variation in selected clinical biomarkers associated with cardiometabolic risk, in response to the consumption of plant-based bioactives (poly)phenols and phytosterols. The present review summarizes the main findings resulting from the meta-analyses already completed.

**Results:**

Meta-analyses of randomized controlled trials conducted within POSITIVe suggest that age, sex, ethnicity, pathophysiological status and medication may be responsible for the heterogeneity in the biological responsiveness to (poly)phenol and phytosterol consumption and could lead to inconclusive results in some clinical trials aiming to demonstrate the health effects of specific dietary bioactive compounds. However, the contribution of these factors is not yet demonstrated consistently across all polyphenolic groups and cardiometabolic outcomes, partly due to the heterogeneity in trial designs, low granularity of data reporting, variety of food vectors and target populations, suggesting the need to implement more stringent reporting practices in the future studies. Studies investigating the effects of genetic background or gut microbiome on variability were limited and should be considered in future studies.

**Conclusion:**

Understanding why some bioactive plant compounds work effectively in some individuals but not, or less, in others is crucial for a full consideration of these compounds in future strategies of personalized nutrition for a better prevention of cardiometabolic disease. However, there is also still a need for the development of a substantial evidence-base to develop health strategies, food products or lifestyle solutions that embrace this variability.

## Key messages

- A balanced diet, rich in plant-based foods is known for the prevention of obesity, diabetes, and cardiovascular disease risk.
- (Poly)phenols and phytosterols displayed a range of biological effects of relevance to contribute to the cardiometabolic health benefits of plant foods
- However, inter-individual variability in response to plant food bioactive consumption exists, and there is a need to understand the causes of this variation.
- Analysis of published RCTs examining impact of consumption of (poly)phenols and phytosterols on cardiometabolic risk factors demonstrated that a number of factors including age, sex, adiposity and health status could contribute to the effect demonstrated within these studies.
- Genome and microbiome studies will help identify what may be causing this variation.
- More studies, specifically designed to investigate individual variation are needed to fully understand the factors responsible for and the impact of this variation
- Once fully understood, such variation should be used in directing personalised nutrition advice.

## Introduction

Many societies struggle with the societal and economic consequences of the rise in cardiometabolic diseases (CMD), including heart disease, stroke and type 2 diabetes mellitus (T2DM*)* [1]. Poor dietary habits are recognized as a major determinant of risk of CMD [2] and focus on the promotion of healthful diets has been identified by policymakers as a cornerstone for public health strategies. From a number of population-based and intervention studies, a consensus has emerged on the beneficial effects of a balanced diet, rich in plant-based foods for the prevention of obesity, diabetes, and cardiovascular disease [3–5]. Thus far, recommendations for plant foods are promoted at a population level in a “one-size fits-all approach”, which does not necessarily ensure that everyone is adequately exposed to and benefit from the protective constituents provided by these foods. In addition to providing low energy and essential micronutrients, plant-based foods are exclusive and abundant sources of a variety of biologically active phytochemicals with known health-promoting effects [6]. These bioactive compounds include (poly)phenols (i.e., flavonoids, phenolic acids, ellagitannins), carotenoids, glucosinolates and phytosterols (plant sterols and stanols), known to display a wide range of biological activities linked to the prevention of a broad range of chronic diseases [7–10]. A growing body of evidence indicates that increased intake of these bioactive compounds, especially (poly)phenols and phytosterols, may help to reduce the risk of CMD [11–13]. For example, the lipid-lowering effects of phytosterols have been extensively studied and reviewed in a meta-analysis, showing that daily consumption of plant sterol-enriched foods lowers total serum and low-density lipoprotein (LDL) cholesterol levels [14]. This effect is mediated by competitive inhibition of cholesterol absorption and transcriptional induction of genes involved in the intestinal and hepatic metabolism of cholesterol [15]. Some physiological effects with implications for cardiometabolic health attributed to (poly)phenols include improvements in endothelial function, platelet function, blood pressure, blood lipid profile and insulin sensitivity [16, 17]. The underlying mechanisms of action are thought to be related to the ability of (poly)phenols to modulate oxidative processes and inflammation regulating cell signaling, insulin resistance, glucose and lipid metabolism amongst other; most of these modulations being mediated by changes in gene expression [18, 19]. More recently, these compounds have been proven to have modulatory properties on the gut microbial ecology with potential repercussions on metabolic health [20, 21].

**Fig. 1:**
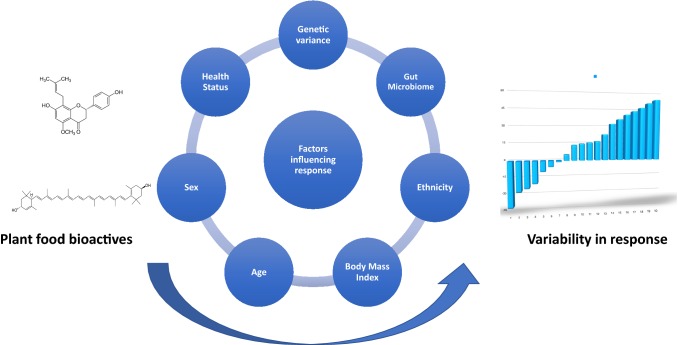
Factors influencing inter-individual variability in cardiometabolic response to plant food bioactive consumption

Research to date has shown that an individual physical/genetic makeup influences their response to dietary interventions, and thereby may influence the benefit/risk associated with consumption of a particular dietary constituent [22]. Whilst still poorly specifically explored, this inter-individual variation in responsiveness is considered to be of particular relevance for dietary plant bioactives [19, 23, 24]. In addition to physical and genetic effects, the influence of the gut microbiota on the biological effect is also of interest, as it is known to be extensively involved in the metabolism of a number of plant bioactives [25]. Together with genetic background and gut microbiome, other factors such as age, sex, lifestyle (diet, smoking, physical activity), ethnicity, pathophysiological status and medication could also be responsible for the heterogeneity in the biological responsiveness to plant food bioactives consumption, and could lead to inconclusive results in some clinical trials aiming to demonstrate the health effects of specific dietary bioactive compounds [26] (Fig. 1).

A clear understanding of why some bioactive plant compounds work effectively in some individuals but not, or less, in others is crucial for a full consideration of these compounds in future strategies of personalized nutrition for a better prevention of CMD in the long run. To address this issue, the European scientific experts involved in the COST Action POSITIVe systematically analyzed data from published studies to assess the inter-individual variation in selected clinical biomarkers associated with cardiometabolic risk, in response to the consumption of plant-based bioactives including flavanols, flavonols, anthocyanins, ellagitannins, and plant sterols. The aim of this review is to summarize the findings obtained regarding identification of potential factors involved in the inter-individual variability in response to (poly)phenols and phytosterols in the context of cardiometabolic disease risk.

## Factors involved in the variability in cardiometabolic response

In each meta-analysis, analyses were first carried out at a total population level, and subsequently at subgroup level (e.g., sex, body mass index (BMI), age, disease, medication, ethnicity) to identify the main factors responsible for between-subject variation beyond bioavailability. The network also performed a systematic analysis of nutrigenomic data available, to identify the cellular and molecular targets involved in the effects of plant food bioactives on cardiometabolic outcomes. The present review summarizes the main findings resulting from this extensive body of work together with the needs and recommendations for future research.

Existing clinical data collected for cardiometabolic risk biomarkers such as blood lipids, blood pressure and BMI show large variations among randomized controlled trials (RCTs) carried out in different countries, from individuals with different lifestyles, ethnicity, age, sex, and physiological/health status, etc. This is further complicated by the variability in the source/form of administration of these plant food bioactives, and duration of the exposure [23, 27]. In this framework, the COST Action FA1403 POSITIVe has delivered, for the first time, focused meta-analyses assessing inter-individual variation in physiological responses linked to selected cardiometabolic endpoints after consumption of plant food bioactives. A key focus of these meta-analyses was to identify (1) groups of the population (stratified by age, sex, ethnicity, BMI, health status, among other parameters) which better respond to (and, therefore, benefit from) the bioactive, and (2) which factors are driving this association beyond genetic polymorphisms and gut microbiota composition. Searches for published RCT were carried out following a registered protocol, stratifying for individual bioactives (registration number: CRD42016037074). This summary will focus on published meta-analyses, examining the effect of flavonols [28], flavanols [29], anthocyanin and ellagitannin-containing products [30] on selected biomarkers of cardiometabolic risk, including blood lipids, blood pressure, endothelial function, glucose homeostasis and anthropometric parameters.

The first meta-analysis examined the effects of various factors on the variability in the responses to their consumption of flavanol-containing tea, cocoa and apple products, where data from 120 RCTs involving 5931 individuals was examined [29]. Selected biomarkers of cardiometabolic risk including body mass index (BMI), waist circumference (WC), total cholesterol (TC), LDL-cholesterol (LDL-c), HDL-cholesterol (HDL-c), and triacylglycerides (TAGs) were considered. Overall, the effects on BMI, WC, total cholesterol and LDL-c appear to be statistically significant in subgroups of people with overweight/obesity (BMI > 25 kg/m^2^). However, there was a lack of evidence to draw conclusions with respect to the influence of certain factors including smoking status, country where the trial was conducted and sex, where the results were inconclusive; with one exception regarding TC which was reduced only in women . [29]. Within this meta-analysis, a smaller selection of trials reported outcome variables linked to glucose homeostasis. Despite the broad range of doses and durations (88–4035 mg flavanols/day; 2–26 weeks), and types of intervention, statistical heterogeneity remained low for these outcomes. The analysis highlighted a consistent small effect on insulin (standardised mean difference) (SMD − 0.25, 95% CI − 0.33; − 0.16) and HOMA-IR ((SMD) − 0.26; 95% CI − 0.36, − 0.16). Subgroup analysis showed lack of effect in those with BMI < 25 or male subjects only, although this may be due to low power, since many of the included trials were unpowered and heterogenous in term of the populations included, with few focusing on very specific groups of the population (sex, or narrow age range) [31].

Similar to flavanol-containing products, the beneficial response to consumption of anthocyanin-rich (berries and red wine, 98 RCTs) and ellagitannin-rich (nuts and pomegranate, 30 RCTs) products intake on TC, diastolic blood pressure (DBP) and systolic blood pressure (SBP) were consistently observed only in people with overweight/obesity [30]. The effect of other factors analyzed such as sex, smoking status, health status or country where the study was conducted were inconsistent across the studies or were noted to require further investigation [30].

The work focusing on RCTs administering flavonols yielded a limited number of trials examining cardiometabolic outcomes [28]. Overall the analysis highlighted the efficacy of flavonol-focused interventions, modestly decreasing TC (− 0.10 mmol/L; 95% CI − 0.20, − 0.01), LDL-c (− 0.14 mmol/L; 95% CI − 0.21, 0.07), TAG (− 0.10 mmol/L; 95% CI − 0.18, 0.03), fasting plasma glucose (− 0.18 mmol/L; 95% CI − 0.29, − 0.08), blood pressure (SBP −4.84 mmHg; 95% CI −5.64, −4.04; DBP −3.32 mmHg; 95% CI − 4.09, − 2.55) and increasing HDL-c (0.05 mmol/L; 95% CI 0.02, 0.07). Stratification by age, sex, country, and health status highlighted a consistent lowering of TAG, TC and LDL-c in participants from Asian countries and decrease in LDL-c in participants with diagnosed disease or dyslipidemia, compared to healthy and normal baseline values. More consistent effect were seen with larger flavonol doses administered (> 200 mg per day), and with pure compounds instead of foods [28].

Finally, the work investigating the effects of phytosterols/phytostanols supplementation on the change in apolipoproteins, including APOA1 and APOB and its ratio, as well as on markers of inflammation and endothelial dysfunction including oxidized LDL-c, flow-mediated dilatation (FMD) and plasminogen activator inhibitor 1 (PAI-1) showed a significant reduction in apo B by 0.07 g/L (95% CI − 0.07, − 0.04). This effect was dependent on the food matrix, being higher for margarine and spreads compared to dairy and other types of food matrix. The highest reduction in apo B was observed among trials giving at least 3 g/day of plant sterols/stanols (− 0.13 g/L; 95% CI − 0.25, − 0.01). Plant stanols-enriched products showed a higher decrease in apo B level (− 0.09 g/L; 95% CI − 0.12; − 0.06) than plant sterols (− 0.03 g/L; 95% CI − 0.04, − 0.02). No differential effects were observed by sex; however, a larger decrease by 0.22 g/L in apo B was observed among studies with younger (< 40 years) population. The analyses exploring the effect of stanols and sterols intakes and apo B/apo A1 ratio showed a 0.07 reduction among studies with fortified dairy products. In addition, a reduction in apo B/apo A1 ratio was observed with the amount of phytosterols/phytostanols between 2 and 3 g/day. Significantly higher reduction in ox-LDL was observed among the studies with plant stanols supplemented margarine and spread comparing to dairy and other type of food matrix as well as among studies with participants of 40–50 years of age (effect size − 3.52; *I*^2^ = 0.00%). No significant effect of plant sterols/stanols enriched food on FMD and PAI-1 was observed (data unpublished).

Another important aspect to consider is the effect of the food matrix on response to interventions. The food matrix has been shown to influence response to nutrition interventions in several foods including plant-based foods [32, 33]. When possible, the impact of the food matrix was considered within the reported meta-analyses, whereby provision of pure compounds versus whole food or response across differing food types was considered, if that data were available. For example, examination of the effect of plant phytosterols was considered in different food matrices, where the authors found the effect was dependent on the food matrix, being higher for margarine and spreads compared to dairy and other types of food matrix (data unpublished) reported that more consistent effects were seen with larger flavonol doses administered (> 200 mg per day), and with pure compounds instead of foods. These findings indicate that a matrix effect is likely, which needs to be further investigated in future specifically designed studies and analyses. Furthermore, factors including dietary patterns, timings of supplementation/intake and study duration should also be considered as potential factors influencing the beneficial effect of plant food bioactives. Variability in the bioactives composition of foods tested among the studies can also be very large. Stricter dietary-controlled trials and standardization of intervention conditions could help to reduce biais that could hamper the identification of factors contributing to inter-individual variability.

Overall, the meta-analyses conducted succeeded in highlighting the state-of-the art in terms of the effects of (poly)phenols and phytosterols on cardiometabolic risk factors, and undertook the large task of reviewing and extracting reported factors which may drive the inter-individual variability in the response to the consumption of these bioactives. The beneficial effect ascribed to (poly)phenols is likely to be complemented by other bioactives and nutrients present in these foods (such as fiber, vitamins).

## Influence of the gut microbiota on the variability in cardiometabolic response

Whilst the meta-analyses focused on demographic, clinical and physical factors influencing variability, a growing body of evidence points to the impact of an individual’s gut microbial community, both in term of composition and function, on the high inter-individual variability in the response to plant food bioactives [21, 24]. The gut microbiota is known to show considerable variation, influenced by variability in enterotypes [34], gut microbiota diversity [35] and quantity of microorganisms [35, 36], and, therefore, the gut microbiota phytochemical metabolites can differ among individuals depending on their gut microbiota composition. For example, in the last decade, inter-individual variability in gut microbiota metabolism of (poly)phenols has been reported for different groups. For some (poly)phenols (hesperidin, isoxantohumol, lignans, and proanthocyanidins), a continuous variation among individuals in the excretion of gut microbiome-derived metabolites has been reported rather than a simple classification into a responder/non-responder group or a specific metabotype [37–40]. For other groups (e.g. isoflavones/equol and ellagic acid/urolithins), there is consistent evidence for the existence of clear metabotypes, defining the presence or absence of specific gut microbiome-derived metabolites that allow the assignment of individuals to specific gut metabotypes [25, 37–41]. However, to date the number of clinical trials assessing cardiometabolic health effects of plant bioactives consumption, while considering inter-individual variability in the gut microbiota composition and functionality, is still very limited. Table 1 shows the limited number of RCTs describing evidence of role of gut microbiota in inter-individual variability in response to plant food bioactives intake related to cardiometabolic outcomes, based on their gut microbiota-derived metabolites (metabotypes) such as equol/non-equol producer metabotypes [42, 43] or urolithin metabotypes [44, 45] or by their specific gut enterotypes (i.e. Bacteroides Prevotella enterotypes [46]. In general, the stratification in these studies has furthered the understanding of the differential response to dietary phenolic compounds and can explain some of the large inter-individual variability observed in the response of individuals to these plant food bioactives reported in previous trials or meta-analyses. Whether the effects are produced by the gut microbiome-derived metabolites or the specific gut microbial community considering the metabolites as biomarkers, or perhaps by a synergistic or additive effect remain unexplored [21, 25].

**Table 1:** Evidence of role of gut microbiota in inter-individual variability in response to plant food bioactives intake related to cardiometabolic outcomes in human studies

Plant food bioactive	i.v. in gut microbiota-derived metabolites	Intervention	Volunteers	i.v. in cardiometabolic outcomes	References
Soy protein containing isoflavones	Equol producers and non-producers	99 mg/day; 1 year	Postmenopausal women (*n* = 202)	Systolic/diastolic blood pressure decreased and endothelial function improved in the equal producers, whereas blood pressure increased and endothelial function deteriorated in the equal non-producers	[42]
Microbial‐derived isoflavone metabolite equol	Equol producers and non-producers	80 mg; acute study (24 h)	Men at moderate cardiovascular risk (*n* = 28)	Arterial stiffness (carotid-femoral pulse-wave velocity) improved in equal producers	[43]
Capsaicin from chili powder	Specific gut enterotypes (Bacteroides enterotype and Prevotella enterotype)	5 and 10 mg/day; 6 weeks	Healthy volunteers (*n* = 12)	Firmicutes/Bacteroidetes ratio, *Faecalibacterium* abundance, plasma levels of glucagon-like peptide 1 and gastric inhibitory polypeptide increased, accompanied with increased and plasma ghrelin level decreased in Bacteroides enterotype. Higher fecal *Faecalibacterium* abundance and butyrate concentration in Bacteroides enterotype	[46]
Pomegranate extract	Urolithin metabotypes A, B, and 0	Dose-1 (160 mg phenolics/day) or dose-2 (640 mg phenolics/day); 3 weeks	Healthy volunteers with overweight/obesity(*n* = 49)	Total cholesterol, LDL-cholesterol, small LDL-cholesterol, non-HDL-cholesterol, apolipoprotein B, and oxidized LDL-cholesterol dose dependently decreased but only in metabotype B individuals	[44]
Red raspberries	Urolithin metabotypes A, B, and 0	Dose-1 (200 g) or dose-2 (400 g); acute study (2 and 24 h)	Healthy males (*n* = 10)	Flow-mediated dilation (FMD) increased in both metabotype A and B, although a non-significant trend towards metabotype A was reported	[45]

## Influence of cell and molecular targets on variability in response: mechanisms of action

Whilst evidence exists of the variability in cardiometabolic response to consumption of plant food bioactives, it is important to both understand the molecular mechanism of action and that of the variability in response. The beneficial health effects of the plant food bioactives have long been attributed solely to their antioxidant activity, however, it is now commonly accepted that their effects are in fact more dependent on their capacity to modulate the expression of genes and proteins or induce epigenetic modifications [47, 48]. For example, anthocyanins prevent the development of atherosclerosis by modulating expression of genes in aorta in ApoE mice [47] and can also modulate expression of genes related to endothelial cell function [19]. Carotenoids have been also shown to be able to modulate the expression of inflammatory-related genes, such as tumor necrosis factor alpha (TNF-alpha), and interleukin 1 beta (IL-1beta) [49]. More recently it has been shown that flavanols can simultaneously modulate difference cell regulatory pathways by affecting not only expression of genes, but also that of proteins and microRNAs, together with changing DNA methylation profiles [50].

To examine the existing evidence, a systematic analysis of the reported genomic effects of specific plant food bioactive compounds was conducted, followed by global bioinformatic analyses of the extracted data to identify key genes underlying their suggested beneficial health properties. Focusing on flavanols, over 100 papers that reported modification in gene expression following exposure to flavonoids from apple, tea, cocoa or grape seed were identified. For in vitro studies, attention was paid to select only publications that have used plant food bioactives at physiologically achievable concentrations, and exclude those using non-physiologically relevant forms and concentrations. Using this approach, about 150 genes with reported expression modulated by flavanols in vitro and in vivo were identified. Among these, TNF-alpha, fatty acid synthase (FASN), monocyte chemoattractant protein-1 (CCL2), interleukin 6 (IL6), peroxisome proliferator-activated receptor alpha (PPAR-alpha) and peroxisome proliferator-activated receptor gamma (PPAR-gamma) were most commonly observed, suggesting that these genes play an important role in the cardiometabolic health properties of flavanols (unpublished data). Once identified, the genes modulated by flavanols were then analyzed for protein–protein interactions, with the aim to identify genes in the nodes of the interaction network. Using this approach, we identified several protein nodes including TNF-alpha, IL6, NDUFAB1, ACACA, TLR4 and P65, some of them having interactions with over 50 proteins. Bioinformatic analyses allowed us further to identify cellular pathways in which differentially expressed genes are involved, which include PPAR-signaling pathway, TNF-signaling pathway, insulin-signaling pathway, leukocyte transendothelial migration and NF-kappa B-signaling pathway (manuscript in preparation). Whilst these results are interesting and will further the understanding of this field, it should be mentioned that there are probably other genes and pathways underlying the health effects of flavanols that were not identified, as most of the studies have used targeted approach and evaluated expression of a few specific genes, thus inducing bias in the interpretation of data. Bioinformatic analyses also allowed us to identify transcription factors involved in the genomic modifications induced by flavanols. Among the most significant ones identified are SP1, PPARa, STAT3, NF-kB and c-myc. The activity of these transcription factors could be modulated by binding of plant food bioactives to them or to cell-signaling proteins and receptors involved in different cellular signaling pathways [19].

Analyses of genomic data by bioinformatic tools allowed us to identify key genes involved in health effects of plant food bioactives, including genes coding for cell-signaling proteins or transcription factors. Due to the key role in cardiometabolic health effects of these bioactives, it could be suggested that polymorphisms in these genes would affect gene-bioactive interaction and consequently biological responsiveness to their intake. Taking TNF-alpha as an example, a search of SNP databases [Variation Viewer (https://www.ncbi.nlm.nih.gov/variation/view/overview) or Ensembl genome database (https://www.ensembl.org/index.html)] showed several hundreds of SNPs in this gene, some of which were insertion or deletion of bases in the DNA, missense mutations, mutations in 5′ UTR region which encompass the promoter region of the gene, frameshift or nonsense/stop gained, with 23 SNPs with a minor allele frequency of > 0.1 or nearly 60 with minor allele frequency > 0.05. Some of these SNPs were previously identified as associated with high or low blood pressure using genome-wide association studies, as identified from genome-wide association studies (GWAS) Catalog database (https://www.ebi.ac.uk/gwas/). Furthermore, several SNPs were observed to be associated with known variability in the effects of certain drugs [as observed using Pharmacogenomics Knowledgebase (https://www.pharmgkb.org/)] and nutrients [51, 52]. Taken together, this strategy of global analysis of genomic effects of plant food bioactives, followed by bioinformatic analyses and search of SNPs databases, allows identification of key genes and polymorphisms potentially involved in inter-individual variability in biological responsiveness to intake of plant food bioactives, which needs to be verified in future nutrigenetic studies.

## Future work and study design

Whilst the work summarized in this review contributes greatly to the scientific evidence supporting the beneficial effect of plant-based bioactives on risk of CMD, it also raises several issues related to the reporting of existing work and future study designs. First in conducting the meta-analyses, we found large discrepancies in reporting quality between the studies considered, with no consensus for population description, or descriptive statistics used. As such there is scope for the nutrition community to adopt a consensus when describing trial participant characteristics, to enable full appraisal of the study findings, with respect to inter-individual variability of response. Welch et al. (2011), as part of an ILSI taskforce, published guidelines for the design, conduct and reporting of human intervention studies to evaluate the health benefits of foods [53]. In this paper, the authors also underlined the importance to consider, and overcome by an adapted study design, the biological variability of the biomarkers measured. This variability, which may have several origins (including among others, genetics, circadian or seasonal variation, female menstrual cycle), can also introduce systematic biais into results [53]. Within the COST Action POSITIVe, this area has been further considered to develop recommendations specifically for the reporting of results which would allow future assessment of factors influencing variation. Addressing the requirement for reporting of results at the onset of a study design will also increase the ability of future studies to be pooled to examine variation in inter-individual variation across and within various population groups, using statistical approaches to identify responders and non-responders [54–56]. The work within the COST Action POSITIVe also highlighted the need to conduct trials within well-defined study populations, in terms of age, sex, ethnicity or health status. Further RCTs, designed to phenotype individuals upon consumption of plant food bioactives are required to fully comprehend the factors affecting inter-individual responses and thereby improve their efficacy in the prevention of cardiometabolic disorders. Conducting this work highlighted the fact that some subgroups of the population receive relatively less research attention, translating to a weaker evidence-base regarding the effectiveness of dietary interventions for some groups compared to others, thus limiting the scope of analyses and interpretation of findings.

Designing specific studies to examine effect both within and between specific groups from the outset will ensure that studies are adequately powered to examine effect of bioactives across specific groups within a population. Such studies are challenging as they will require screening and/or targeted recruitment, for a specific phenotypic or genotypic characteristic, but will add significantly to the evidence base that will allow idenfication of factors influencing inter-individual variability. This approach is time-consuming but allows the correct interpretation of factors influencing response. For example, work examining the nutrient–gene interaction of methylene tetra-hydrofolate reductase (MTHFR) and vitamin B2 (riboflavin) consumption, using a targeted recruitment approach, elucidated the fact that riboflavin supplementation was successful in reducing BP in hypertensive individuals with the MTHFR 677TT genotype only (compared to MTHFR 677CC, or CT genotypes), more effectively than pharmacological treatment indicating the potential for a personalized approach to the management of hypertension in this genetically at-risk group [57].

## What do these findings mean for clinicians, nutritionists, and food industry?

The translational value of the work carried out by the COST Action POSITIVe has important implications for public health, clinical nutrition and the food industry. At present, the emerging evidence highlights the preventive, and sometime therapeutic, potential of (poly)phenols intake—with effect size ranging from small to moderate on markers of CMD. Of particular interest, our findings confirm that major factors shape the inter-individual variability in the response to (poly)phenolics—these included sex, BMI and baseline cardiometabolic markers, among others. However, these findings need cautious interpretation. Such findings are based only on existing published data, where gaps were identified, for example the degree of characterisation of the gut microbiota, or even bioavailability. Furthermore, the findings were consistent in groups we would expect to benefit, for example people with overweight/obesity and those clinically compromised. Although we see response in such groups, the contribution of these factors is not yet demonstrated consistently across all (poly)phenols subclasses and cardiometabolic outcomes, partly due to the heterogeneity in trial designs, low granularity of data reporting, variety of food vectors (and food matrices) and target populations, and potentially through biological mediation of responses.

We anticipate that, as the field evolves and implements more stringent reporting practices, factors identified influencing response could guide differential dietary advice in subgroups that would benefit most. Issues with respect to regulatory aspects, including labeling and health claims need to be addressed, however. There is also still a need for the development of a substantial evidence-base to develop health strategies, food products or lifestyle solutions that embrace this variability and will be accepted by consumers and health care professionals [58]. Nonetheless, this area could prove to be an important intersection between industry and public health, since it has been demonstrated that personalization results in better engagement than generic advice [59].

However, before the physiological and demographic factors identified in the COST Action’s meta-analyses can be applied in the further development of personalized nutritional advice or targeted products in the market, there is a need to determine the extent in which these factors contribute to inter-individual variation compared to behavioral and other contextual factors. In addition, there is a need for adequately powered studies (and/or individual data meta-analyses) to confirm the differing impact of the consumption of plant food bioactives within specific population groups, identified in the Action’s meta-analyses on inter-individual variation in responses to bioactives.

Overall, the findings and approach taken within the work summarized here demonstrates that inter-individual variation does influence the response to consumption of food bioactive compounds. If properly understood this could be used for targeted or personalized recommendations and/or development of food products for specific population groups.

## Abbreviations

APOA1: Apolipoprotein A1
APOB: Apolipoprotein B
ApoE: Apolipoprotein E
BMI: Body mass index
BP: Blood pressure
CCL2: Monocyte chemoattractant protein-1
CI: Confidence interval
CMD: Cardiometabolic disease
DNA: Deoxyribonucleic acid
FASN: Fatty acid synthase
FMD: Flow-mediated dilation
GWAS: Genome-wide association studies
HDL: High-density lipoprotein
HDL-c: HDL-cholesterol
HOMA-IR: Homeostatic model assessment of insulin resistance
IL6: Interleukin 6
LDL-c: LDL-cholesterol
LDL: Low-density lipoprotein
MTHFR: Methylene tetra hydro folate reductase
PAI-1: Plasminogen activator inhibitor 1
PPAR-alpha: Peroxisome proliferator-activated receptor alpha
PPAR-gamma: Peroxisome proliferator-activated receptor gamma
RCT: Randomized controlled trial
SMD: Standardized mean difference
SNP: Single-nucleotide polymorphism
T2DM: Type 2 diabetes mellitus
TAG: Triacylglyceride
TC: Total cholesterol
UTR: Untranslated region
WC: Waist circumference
